# *Episyrphus balteatus* symbiont variation across developmental stages, living states, two sexes, and potential horizontal transmission from prey or environment

**DOI:** 10.3389/fmicb.2023.1308393

**Published:** 2024-01-05

**Authors:** Xiao Chang, Shuang Xue, Ruimin Li, Yuanchen Zhang

**Affiliations:** ^1^National Key Laboratory of Cotton Bio-Breeding and Integrated Utilization, Institute of Cotton Research, Chinese Academy of Agricultural Science, Anyang, Henan, China; ^2^School of Biological and Food Engineering, Anyang Institute of Technology, Anyang, China; ^3^Research Base, Anyang Institute of Technology, National Key Laboratory of Cotton Bio-breeding and Inte-grated Utilization, Anyang, Henan, China; ^4^Taihang Mountain Forest Pests Observation and Research Station of Henan Province, Linzhou, China

**Keywords:** marmalade hoverfly, *Megoura crassicauda*, symbionts, health and dying adult, 16S rRNA sequencing, horizontal transmission

## Abstract

**Introduction:**

*Episyrphus balteatus* is one representative Syrphidae insect which can provide extensive pollination and pest control services. To date, the symbiont composition and potential acquisition approaches in Syrphidae remain unclear.

**Methods:**

Herein, we investigated microbiota dynamics across developmental stages, different living states, and two sexes in *E. balteatus* via full-length 16S rRNA genes sequencing, followed by an attempt to explore the possibility of symbiont transmission from prey *Megoura crassicauda* to the hoverfly.

**Results:**

Overall, Proteobacteria and Firmicutes were the dominant bacteria phyla with fluctuating relative abundances across the life stage. *Cosenzaea myxofaciens* is dominant in adulthood, while *Enterococcus silesiacus* and *Morganella morganii* dominate in larvae and pupae of *E. balteatus*, respectively. Unexpectedly, *Serratia symbiotica*, one facultative endosymbiont commonly harbored in aphids, was one of the predominant bacteria in larvae of *E. balteatus*, just behind *Enterococcus silesiacus*. In addition, *S. symbiotica* was also surprisingly most dominated in *M. crassicauda* aphids (92.1% relative abundance), which are significantly higher than *Buchnera aphidicola* (4.7% relative abundance), the primary obligate symbiont of most aphid species. Approximately 25% mortality was observed among newly emerged adults, of which microbiota was also disordered, similar to normally dying individuals. Sexually biased symbionts and 41 bacteria species with pairwise co-occurrence in *E. balteatus* and 23 biomarker species for each group were identified eventually. Functional prediction showed symbionts of hoverflies and aphids, both mainly focusing on metabolic pathways. In brief, we comprehensively explored the microbiome in one Syrphidae hoverfly using *E. balteatus* reared indoors on *M. morganii* as the model, revealed its dominated symbiont species, identified sexually biased symbionts, and found an aphid facultative endosymbiont inhabited in the hoverfly. We also found that the dominated symbiotic bacteria in *M. crassicauda* are *S. symbiotica* other than *Buchnera aphidicola*.

**Discussion:**

Taken together, this study provides new valuable resources about symbionts in hoverflies and prey aphids jointly, which will benefit further exploring the potential roles of microbiota in *E. balteatus*.

## 1 Introduction

Insects are the most abundant and diverse species in the animal kingdom with numerous essential ecological functions in the biosphere. The evolutionary success of insects in adapting to various environmental conditions, diets, and habitats is inseparable from the contribution of their symbiotic microbes (Ali et al., 2019). These various microorganisms are usually colonized on/in the exoskeletons, intestines, blood cavity, salivary glands, and even individual cells, accounting for 1%–10% of insect biomass (Zhao et al., 2022). Symbiotic bacteria act as important regulators of host insect’s multiple lifestyles and participate in the tri-interaction of plants, insects, and natural enemies, including but not limited to food consumption and digestion, defense against various abiotic/biotic stressors (i.e., predators, pathogens, parasites, heat, etc.), reproduction (adult mating, sex ratio of offspring), provision of amino acids and nutrients, and insecticide resistance (Xue et al., 2021; Chen et al., 2022; Wang Y. B. et al., 2022; Yao et al., 2023).

Describing the microbial community composition and clarifying the microbiota acquisition and transmission approach contribute greatly to understand how insects and microorganisms associate and mutually benefit from each other. Recently, with the development of 16S rRNA gene sequencing, the microbiota biodiversity and variation have been revealed in considerable insect categories, including but not limited to Lepidoptera (Paniagua Voirol et al., 2018), Hemiptera (Sudakaran et al., 2017), Coleoptera (Salem and Kaltenpoth, 2022), Hymenoptera (Treanor et al., 2018), Diptera (Raza et al., 2020), and Blattaria (Arora et al., 2022). As for microbiota acquisition and transmission, vertical transmission is considered the primary way in most insects by which symbiotic microorganisms are commonly transferred transovarially from mother to offspring within female germ cells (Szklarzewicz and Michalik, 2017; Shan et al., 2021; Wan et al., 2023). Such vertical transfer mode promotes the strong stability for the symbiosis maintaining of microbiota and insects, favoring the evolution of microbially mediated effects that improve host insect fitness (Russell and Moran, 2005). In addition, plentiful evidence indicates that symbionts can also undergo horizontal transfer among different insects or be acquired directly from the environment or diets (Hosokawa et al., 2016; Tzuri et al., 2021; Du et al., 2022; Pons et al., 2022), and acquisition of novel symbiont strains for the insects via horizontal transmission can provide fitness benefits to the host, with significant ecological and evolutionary consequences (Łukasik et al., 2015). In contrast to considerable studies about microbiota structure and diversity in aphids (Guo et al., 2017), bees (Steffan et al., 2023), flies (Noman et al., 2020), bugs (Gonella et al., 2020), and beetles (Salem and Kaltenpoth, 2022), knowledge about symbionts in hoverflies was rare.

Hoverflies, the important pollinators and predators, provide dual ecosystem services including pest control and pollination. To date, plentiful studies exploring genome (Yuan et al., 2022), reproduction (Putra et al., 2009), behavior (Vosteen et al., 2018), and phylogenetic relationships (Wong et al., 2023) have been performed on various hoverfly species. However, studies related to the community and sources of symbionts in hoverflies are few, except for only several bacteria gene identification reports from Pons (Pons et al., 2022) and Sánchez-Galván (Sánchez-Galván et al., 2017). Sánchez-Galván identified three bacteria species genes in the gut of the hoverfly *Mallota dusmeti* via PCR amplification (Sánchez-Galván et al., 2017). Pons found that several genes of symbiont *Serratia symbiotica* can be identified in two hoverfly species (Pons et al., 2022). Except for insect species mentioned above, symbiont diversity has also been systematically revealed in numerous other aphid predators or pollinators such as lacewing flies (Zhao et al., 2019), lady beetles (Gao et al., 2021), and honeybees (Crotti et al., 2013; Lang et al., 2023). Hence, it is urgent to explore the microbiota in Syrphidae insects, including their community, potential acquisition and transmission approach, and even function.

*Episyrphus balteatus*, one long-range migratory hoverfly, transports billions of pollen grains, consumes trillions of aphids, and makes billions of flower visits on the annual fluxes (Wotton et al., 2019; Wang Z. et al., 2022). Considering that the populations of many pollinators, especially bees, are seriously declining (Biesmeijer et al., 2006; Powney et al., 2019), *E. balteatus* is becoming more important in the ecosystem and environment, due to its traveling among high- and low-latitude regions seasonally. However, the different feeding habits of larvae and adults commonly lead to a relatively higher mortality of newly emerged adults or a low pupation rate (Lillo et al., 2021), probably caused by a failure in adapting the transition of feeding habits from aphid feeding to pollen/nectar feeding after eclosion. Tens of thousands of migrating *E. balteatus* were previously reported dead on a strandline in the south of France without any warning (Fisler and Marcacci, 2022). In addition, high mortality also existed at the larval stage of some hoverfly species such as *Eristalinus aeneus* (Campoy et al., 2022). It is speculated that the establishment, maintenance, and refreshing of symbiosis with bacteria for hoverflies are important and dynamic. Various pathogenic or defensive bacteria colonized in aphids, polluted water, dead leaves, or flowers likely impact adversely on aphidophagous, herbivorous, and saprophagous hoverflies. In addition, the transition in feeding habits to consume pollen or nectar in adulthood also requires refreshing their symbiosis with symbiotic bacteria.

To determine the microbial community composition and explore the microbiota acquisition and transmission approach in Syrphidae insects, *Episyrphus balteatus* was chosen as a model in this study. Considering that insect-associated microbiome can vary both between and within species, and intraspecific microbiome variation was influenced by several factors, in which diet and environment appear to be the most relevant drivers (Lange et al., 2023), a pair of marmalade hoverfly adults captured in the field were reared on *M. crassicauda* aphids in the laboratory for more than 10 generations before sampling The microbiota of *E. balteatus* is probably inconstant and unstable due to uncontrollable conditions including temperature, humidity, nutriment (prey/aphid species, pollen from which plant species), and even parasites. Then, the microbiota structure and diversity of *E. balteatus* across different developmental stages (larvae, pupae, adults), living states (healthy adults, normally dying adults, newly emerged dying adults), and two sexes were identified via 16S rRNA genes sequencing. Meanwhile, the mortality of *E. balteatus* newly emerged adults within 3 days after the emergence was evaluated. Furthermore, we analyzed and identified sexually biased symbionts, explored the pairwise co-occurrence of microbial species, and identified biomarkers for each group. To explore the potential of achieving approach of symbionts for *E. balteatus*, microbiota in its prey *M. crassicauda* were investigated as well. Finally, the functions of symbionts of hoverflies and aphids were predicted. Overall, we found that *Cosenzaea myxofaciens*, a bacteria species rarely seen in other insects, is the dominant symbiont in *E. balteatus*; we also identified numerous sexually biased symbionts and microbial species, exploring pairwise co-occurrence relationships, and discussed the potentially horizontal transmission of symbionts from aphids or the environment to the hoverfly.

## 2 Materials and methods

### 2.1 Insect rearing

The marmalade hoverfly adults, captured originally in the field of Taihang Mountain Forest Pests Observation and Research Station of Henan Province (Linzhou, China), were reared in the laboratory for more than 10 generations under the condition of 21°C ± 1°C with (60 ± 5) % relative humidity and a photoperiod of 14-h light:10-h dark. The larvae were fed with *M. crassicauda* aphids on broad bean *Vicia faba*, and emerging adults were provided with maize pollen and 2% sucrose solution. In addition, the mortality of newly emerged female and male hoverfly adults within 3 days was recorded, respectively.

### 2.2 Specimen collection

For *E. balteatus*, whole bodies of nymphs (first to third instar), pupae, normal 3-day-old female and male adults (defined as F^N^ and M^N^, respectively), abnormally dying female and male adults of newly emerged within 3 days (defined as F^AD^ and M^AD^, respectively), and normally dying female and male adults (usually at 15 to 17 days post-emergence, defined as F^ND^ and M^ND^, respectively) were collected, respectively. There were five biological replicates for each developmental stage of *E. balteatus*, and each biological replicate contained at least 50 hoverfly individuals. For aphids, pooled *M. crassicauda* at different developmental stages (first instar nymph to adult) were collected, with five biological replicates. Each replicate comprised 50 to 100 individuals at each developmental stage.

### 2.3 DNA extraction, PCR amplification, and sequencing

Total genomic DNA was extracted from aphids and hoverfly samples using the TGuide S96 Magnetic Soil/Stool DNA Kit (Tiangen Biotech, Beijing), according to the manufacturer’s instructions, respectively. The DNA quality was examined through electrophoresis on a 1.8% agarose gel, and the quantity of DNA was determined using a spectrophotometer NanoDrop 2000 (Thermo, United States). The full-length 16S ribosomal RNA genes of V1-V9 regions were amplified using primer pairs 27F: 5’-AGRGTTTGATYNTGGCTCAG-3′ and 1492R: 5’-TASGGHTACCTTGTTASGACTT-3′.

The PCR amplifications, purification, and quantification of PCR products, library preparation, and sequencing were conducted as previously reported (Xu et al., 2023). The normalized equimolar concentrations of amplicons were pooled and sequenced on the PacBio Sequel II platform (Biomarker, Beijing). The raw reads were submitted to the NCBI Sequence Read Archive (SRA) database with an accession number PRJNA1020272.

### 2.4 OTU clustering

The generated raw reads were filtered and demultiplexed using SMRT Link software (version 8.0) with minPasses ≥ 5 and minPredictedAccuracy ≥ 0.9, aiming to obtain the circular consensus sequencing (CCS) reads. CCS reads containing no primers and those reads beyond the length range (1,200–1,650 bp) were discarded through quality filtering software Cutadapt (version 2.7; Marcel, 2011). Subsequently, the UCHIME algorithm (v8. 1; Edgar et al., 2011) was used to obtain the clean reads by detecting and removing chimeric sequences. Eventually, sequences with similarity >97% were clustered into the same operational taxonomic unit (OTU) by USEARCH (v10.0; Edgar, 2010), and the OTU counts less than 2 in all samples were filtered.

### 2.5 Bioinformatics analysis

The bioinformatics analysis in this study was performed with the aid of the BMKCloud.^1^ Taxonomy annotation of the OTUs was performed based on the Naive Bayes classifier in QIIME2 (Bolyen et al., 2019) using the SILVA database (release 138.1; Quast et al., 2013) with a confidence threshold of 70%. The α-diversity indices of Chao1, Shannon, Simpson, ACE, and OTU numbers were calculated to evaluate the microbial community richness and diversity by the QIIME2 software. β-diversity was determined to evaluate the degree of similarity of microbial communities from different samples using QIIME2.

Principal component analysis (PCA), non-metric multidimensional scaling (NMDS), heatmaps, clustering, and unweighted pair group method with arithmetic mean (UPGMA) were used to compare beta diversity among different samples, using the python package (*sklearn*, *nmds.py*, *ete3*) and R package (*pheatmap*). The Wilcoxon test was utilized to investigate community composition differences with the Benjamini-Hochberg (BH) FDR-corrected *p-*value. Venn diagrams were drawn to visualize unique and shared bacteria across the samples evaluated. Pairwise bacteria with co-occurrence patterns at genus and species levels were performed using Spearman’s analysis (correlation coefficient threshold: ±0.5; *p* < 0.5) and visualized through serial R packages (psych-v2.1.9, igraph-v1.2.5, visNetwork-v2.1.0). Furthermore, the linear discriminant analysis (LDA) effect size (LEfSe; Segata et al., 2011) was employed to identify biomarkers with significant differences among different groups with the logarithmic LDA score of 4.0 as the threshold for discriminative features. To explore the dissimilarities of the microbiome among different factors, a redundancy analysis (RDA) was performed in R using the package *vegan* (v2.3–0). The functions of symbiotic bacteria in aphids and hoverflies were predicted using the software PICRUSt2, the abundance of each functional category was calculated, and the significant differences were explored according to previous methods (Chen et al., 2022).

## 3 Results

### 3.1 Overview of microbiota across *Megoura crassicauda* and *Episyrphus Balteatus*

**Figure 1:**
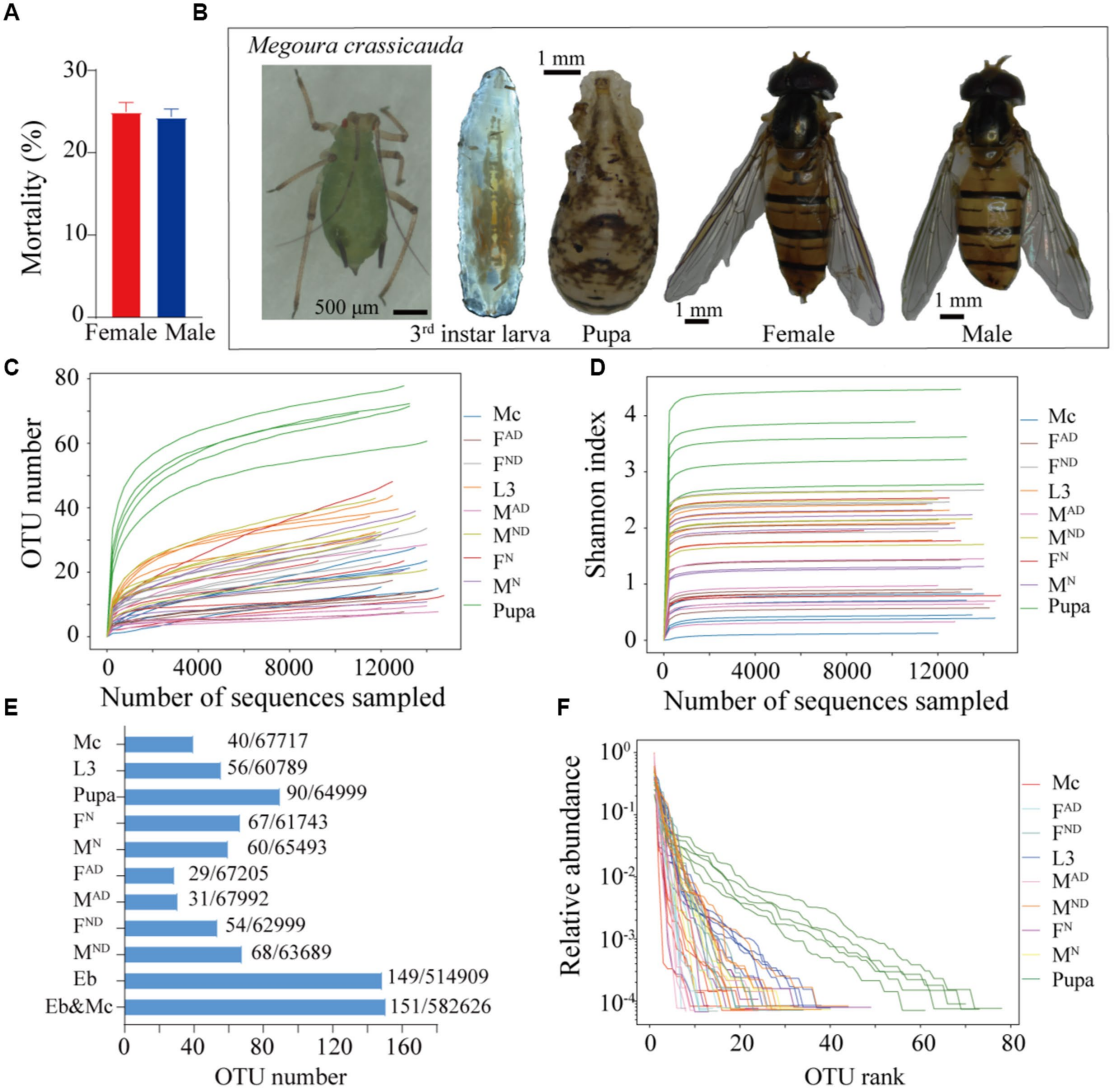
Overview of microbiota dynamics in *Episyrphus balteatus* and *Megoura crassicauda*. The mortality of newly emerged hoverflies within 3 days **(A)**. *M. crassicauda*, larva, pupa, and female and male adults of *E. balteatus* **(B)**. Rarefaction curves of all samples **(C)**. Shannon index dynamics across the sequencing depth **(D)**. The number of OTU and sequence comparison among all samples **(E)**. The rank abundance curves at the OTU level **(F)**. Mc, *M crassicauda*; L3, first to third instar larva of hoverfly; pupa, hoverfly pupa; F^N^ and M^N^, normal 3-day-old female and male hoverfly adult; F^AD^ and MD^AD^, abnormally dying newly emerged female and male hoverflies within 3 days; F^ND^ and M^ND^, normally dying female and male hoverflies after 15 to 17 days post-eclosion.

In our long-term indoor rearing process, approximately 24.3% of male and 24.9% of female adults usually died within 3 days post-eclosion (Figure 1A). Compared to the carnivorous larvae, the vegetarian adults of *E. balteatus* need to establish a new microbiota community after emergence, which probably leads to high mortality of newly emerged adult individuals that fails in refreshing symbiotic bacteria colonization at the earlier adult stage. Hence, the symbiont communities and dynamic changes in *E. balteatus* across developmental stages (larvae, pupae, and adults), different living states, two sexes, and potential horizontal transmission from *M. crassicauda* aphids were investigated (Figure 1B).

A total of 597,744 clean reads which belonged to 151 OTUs were obtained (Figure 1E), in which each sample contained 13,283 CCS clean reads on average (Supplementary Table S1). As for the quality of sequencing, both rarefaction curves (Figure 1C) and Shannon index dynamics (Figure 1D) gradually tended to saturate after the number of sequences reached 2,000, indicating that most microbial species were captured in all samples. Specially, up to 90 OTUs were identified in pupae of *E. balteatus*, followed by 67 and 60 OTUs in F^N^ and M^N^ hoverfly adults, 54 and 68 OTUs in FD^N^ and MD^N^ hoverfly adults, and 56 and 40 OTUs in hoverfly larvae and *M. crassicauda* aphids, respectively. As expected, FD^3DPE^ and MD^3DPE^ hoverfly adults owned the least OTU numbers, 29 and 31, respectively (Figure 1E). These identified OTUs were clustered into 10 phyla, 13 classes, 34 orders, 59 families, 98 genera, and 137 species (Supplementary Table S2). In addition, the rank abundance curves also showed that pupae of hoverflies owned most bacterium species with a higher heterogeneous distribution than other samples (Figure 1F).

**Figure 2:**
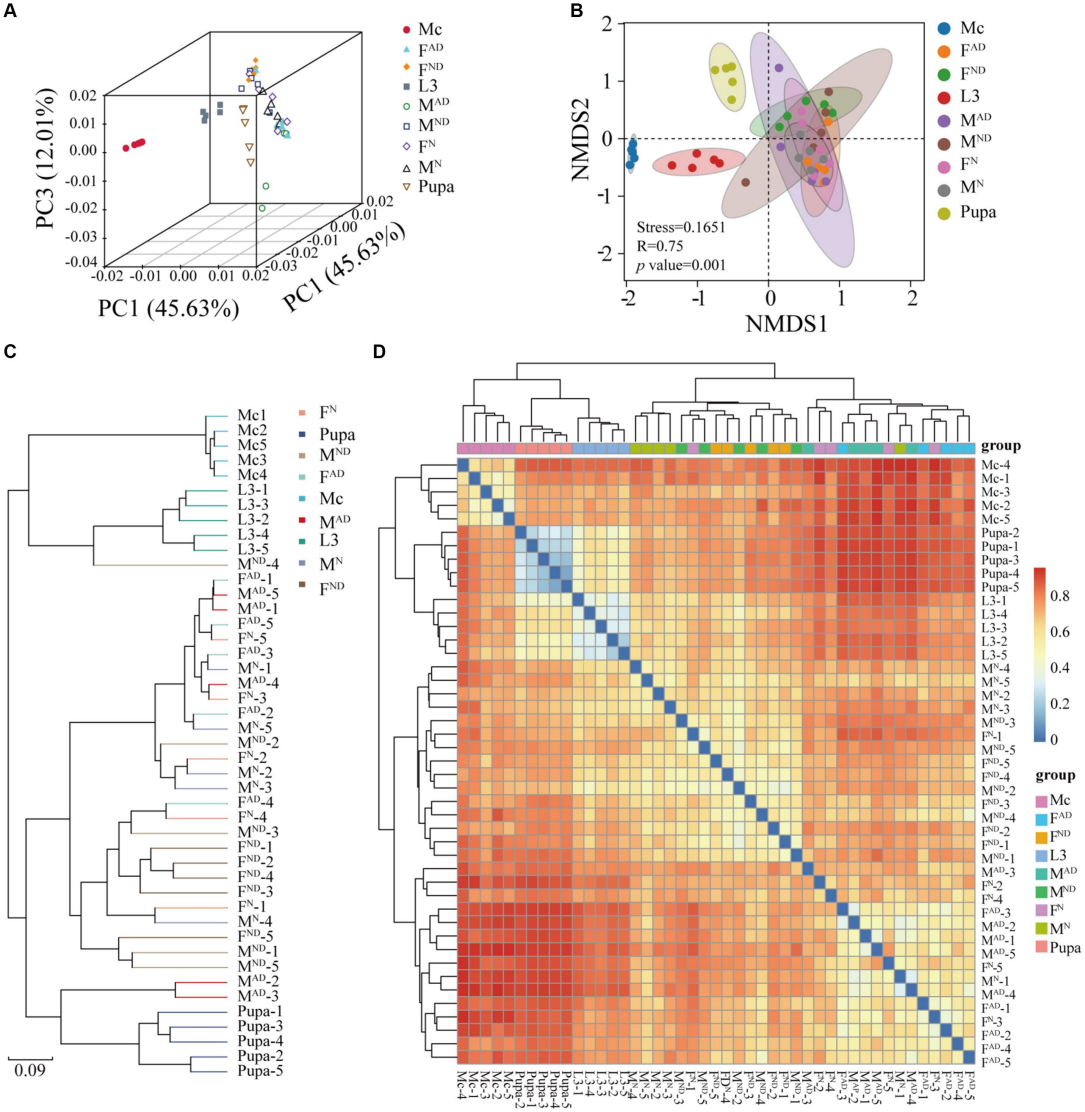
Sample clustering analysis of PCA **(A)**, NMDS **(B)**, UPGMA **(C)**, and heatmap clustering **(D)**. Principal component analysis (PCA) plots **(A)** at the OTU level and NMDS plots **(B)** and the species level based on Bray–Curtis distances of symbiont communities across all groups. In DMDS plots, analysis of similarities (ANOSIM) was used to obtain the *p*-value (i.e., significance levels) and an R-value (i.e., the strength of the factors on the samples). R-value is supposed to vary between 0 and 1. R-value close to 1 indicates high separation among groups, while R-value close to 0 indicates no separation. Finally, usually stress value< 0.2 suggests the realizable analysis results. Unweighted pair-group method with arithmetic means (UPGMA) analysis of bacterial community structure based on Bray–Curtis distances at the species level **(C)**. Heatmap of major taxa across samples at the OTU level. Cluster analysis using the binary_Jaccard distance and the complete-linkage method. Plotting scale, from red to blue, indicates the decrease in correlation. Five replicates are labeled 1–5. The definition of Mc, L3, F^N^ and M^N^, F^AD^ and M^AD^, F^ND^, and M^ND^ was the same as above in Figure 1.

The PCA (Figure 2A), NMDS (Figure 2B), UPGMA (Figure 2C), and heatmap clustering analysis (Figure 2D) results demonstrated that samples from *M. crassicauda* aphids, hoverfly larvae, and pupae exhibited significant separation, while the variability was also low between different replicates within the same groups. In contrast, six hoverfly adult samples clustered together with a higher variability within the same group, which is probably due to the complexity and flexibility of the bacterial community of adult hoverflies caused by larva–adult diet styles and feeding habit changes (carnivorous/chewing to vegetarian/sucking).

### 3.2 Microbiota comparison between *Megoura crassicauda* and *Episyrphus Balteatus*

Taxonomic analysis showed that Proteobacteria were the most prevalent phylum in all aphid and hoverfly samples (Supplementary Figure S1A; Supplementary Tables S3, S4). At the class level, Gammaproteobacteria was the most dominant with the highest relative abundance of 97.7% in *M. crassicauda* aphids, followed by 34.3%–91.9% relative abundance in adult hoverflies, 55.9% relative abundance in hoverfly larvae, and 35.3% relative abundance in hoverfly pupae. Interestingly, in all hoverfly groups (not including pupae), the second and third dominant classes were Alphaproteobacteria and Bacilli, whereas the second and third dominant classes in aphids and hoverfly larvae were Bacilli and Actinobacteria, and in hoverfly pupae, the second and third classes were Bacilli and Bacteroidia (Supplementary Figure S1B; Supplementary Tables S3, S4). At the order level, Enterobacterales is the most dominant in all groups with a relative abundance of 59.1%–97.1%. However, its relative abundance in pupae (25.8%) and F^DN^ of hoverflies (34.1%) was low, with the dominant bacteria belonging to Staphylococcales (30.0%) and Rickettsiales (39.1%), respectively (Supplementary Figure S1C; Supplementary Tables S3, S4). At the family level, Yersiniaceae is the most dominant in aphids and hoverfly larvae, while in F/M^N^ (normal 3-day-old female and male adults) and F/M^AD^ (abnormally dying female and male adults of newly emerged within 3 days) hoverflies, the most predominant bacteria family was Morganellaceae. However, in hoverfly pupae and F/M^ND^ (normally dying female and male adults) hoverfly adults, the most dominant bacteria families were Staphylococcaceae, Anaplasmataceae, and Hafniaceae, respectively (Supplementary Figure S1D; Supplementary Tables S3, S4).

**Figure 3:**
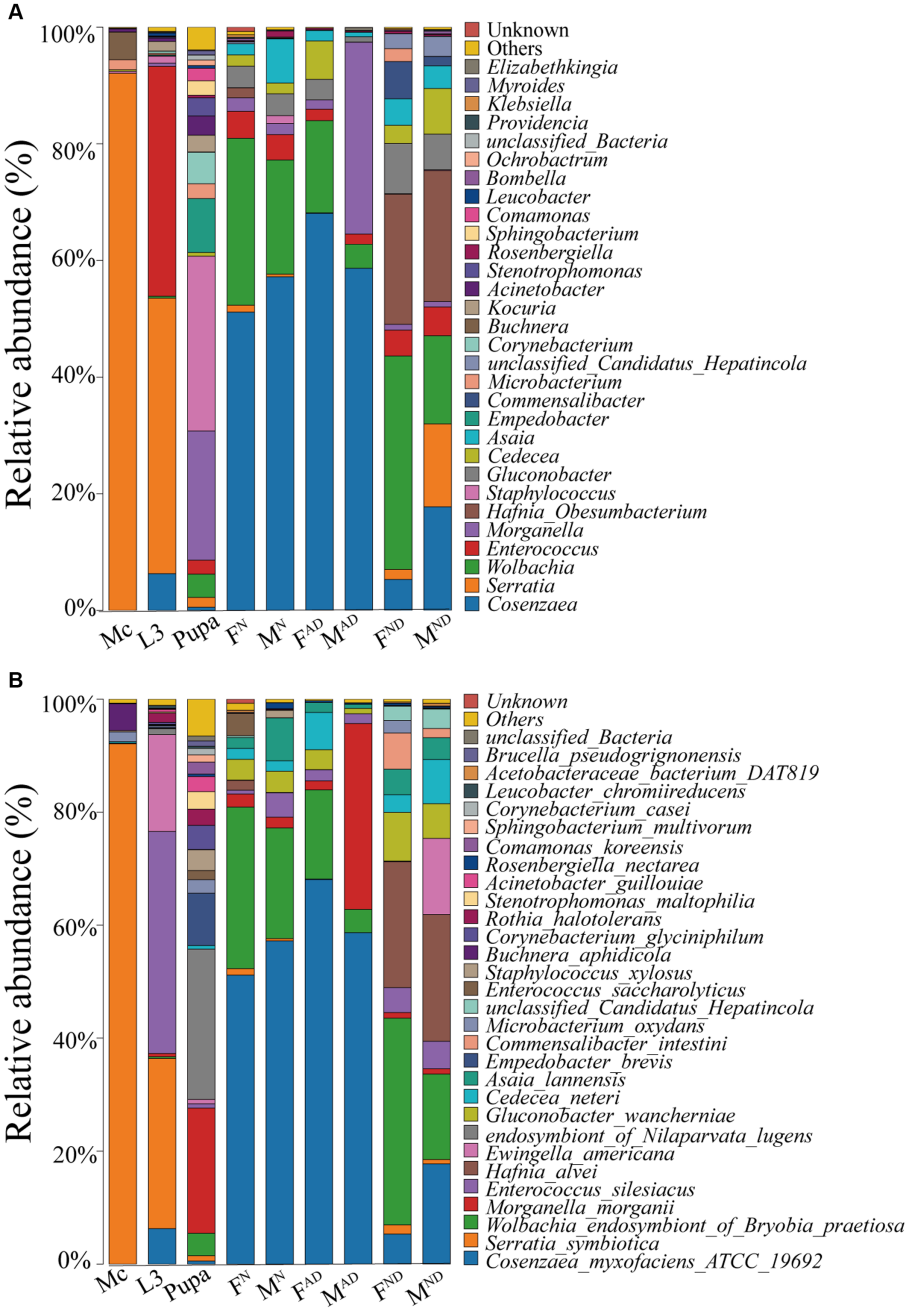
Symbiont community dynamics among aphids and hoverflies. Relative abundance of symbiont communities in different groups at the phylum **(A)** and species levels **(B)**. The definition of Mc, L3, F^N^ and M^N^, F^AD^ and M^AD^, F^ND^, and M^ND^ was the same as above in Figure 1.

Interestingly, at the genus level, *Serratia* is the most dominant in aphids and hoverfly larvae with a relative abundance of 92.1% and 47.2%, respectively, followed by *Buchnera* and *Microbacterium* in aphids with a relative abundance of 4.7 and 1.7%, and *Enterococcus* and *Cosenzaea* in hoverfly larvae with the relative abundance of 39.4% and 6.3%, respectively (Figure 3A; Supplementary Table S3, S4). However, in F/M^N^ and F/M^AD^ hoverfly adults, the most dominant genus was *Cosenzaea* (51.2%–68.1% relative abundance), followed by Wolbachia (15.8%–28.6% relative abundance in F/M^DN^ and F^AD^) and Enterococcus (32.9% relative abundance in M^AD^). In hoverfly pupae, *Staphylococcus* (30.0% relative abundance) and *Morganella* (22.1%) were the dominant bacteria genera followed by *Empedobacter* (9.3%), *Corynebacterium* (5.4%), and *Wolbachia* (4.0%), while in F/M^ND^ hoverfly adults, *Wolbachia* (36.6% in F^ND^ and 15.1% in M^ND^), *Hafnia-Obesumbacterium* (22.3% in F^ND^, 22.4% in M^ND^), *Cosenzaea* (5.3% in F^ND^, 17.8% in M^ND^), *Gluconobacter* (8.6% in F^ND^), and *Serratia* (14.2% in M^ND^) were the top dominant bacteria (Figure 3A; Supplementary Tables S3, S4).

At the species level, the dominant bacteria species in *M. crassicauda* aphid was *Serratia symbiotica*, with a relative abundance of 92.1%, other than *Buchnera aphidicola* (4.7%; Figure 3B; Supplementary Tables S3, S4), one familiar primary symbiotic bacterium existed in most aphid species. Interestingly, in hoverfly larvae, aphid endosymbiotic bacterium *S. symbiotica* remains one of the dominant bacterial species with a relative abundance of 30.1%, second only to *Enterococcus silesiacus* (39.3%), followed by *Ewingella americana* with 17.1% relative abundance. In most hoverfly adults (not including normally dying adults), the most dominant bacteria species was *Cosenzaea myxofaciens_ATCC_19692* (51.2%–68.1%), followed by *Wolbachia endosymbiont_of_Bryobia_praetiosa* and *Morganella morganii* (Figure 3B; Supplementary Tables S3, S4). In contrast, for the hoverfly pupae, the dominant bacteria species were *endosymbiont_of_Nilaparvata_lugens* and *Morganella morganii*, whereas in F^ND^ and M^ND^ hoverfly adults, the dominant bacteria species were *Wolbachia_endosymbiont_of_Bryobia_praetiosa*, *Hafnia alvei*, and *Cosenzaea_myxofaciens_ATCC_19692*.

To some extent, the bacterial community of *M. crassicauda* aphids and *E. balteatus* larvae was more similar (Figure 3; Supplementary Figure S1). In addition, normally dying hoverfly adults and abnormally dying adults of newly emerged own some similar bacteria community (Figure 3; Supplementary Figure S1), which is probably due to the fact that they were all just adults collected within 3 days post-emergence but with different living states, namely, vigorous or dying. In addition, to some degree, normally dying female and male hoverfly adults were similar in the bacterial community as well. Interestingly and unexpectedly, the bacterial community of hoverfly pupae was the most special and different from other hoverfly and aphid samples (Figure 3; Supplementary Figure S1).

### 3.3 Symbiont horizontal transmission from *Megoura crassicauda* to *Episyrphus balteatus*

**Figure 4:**
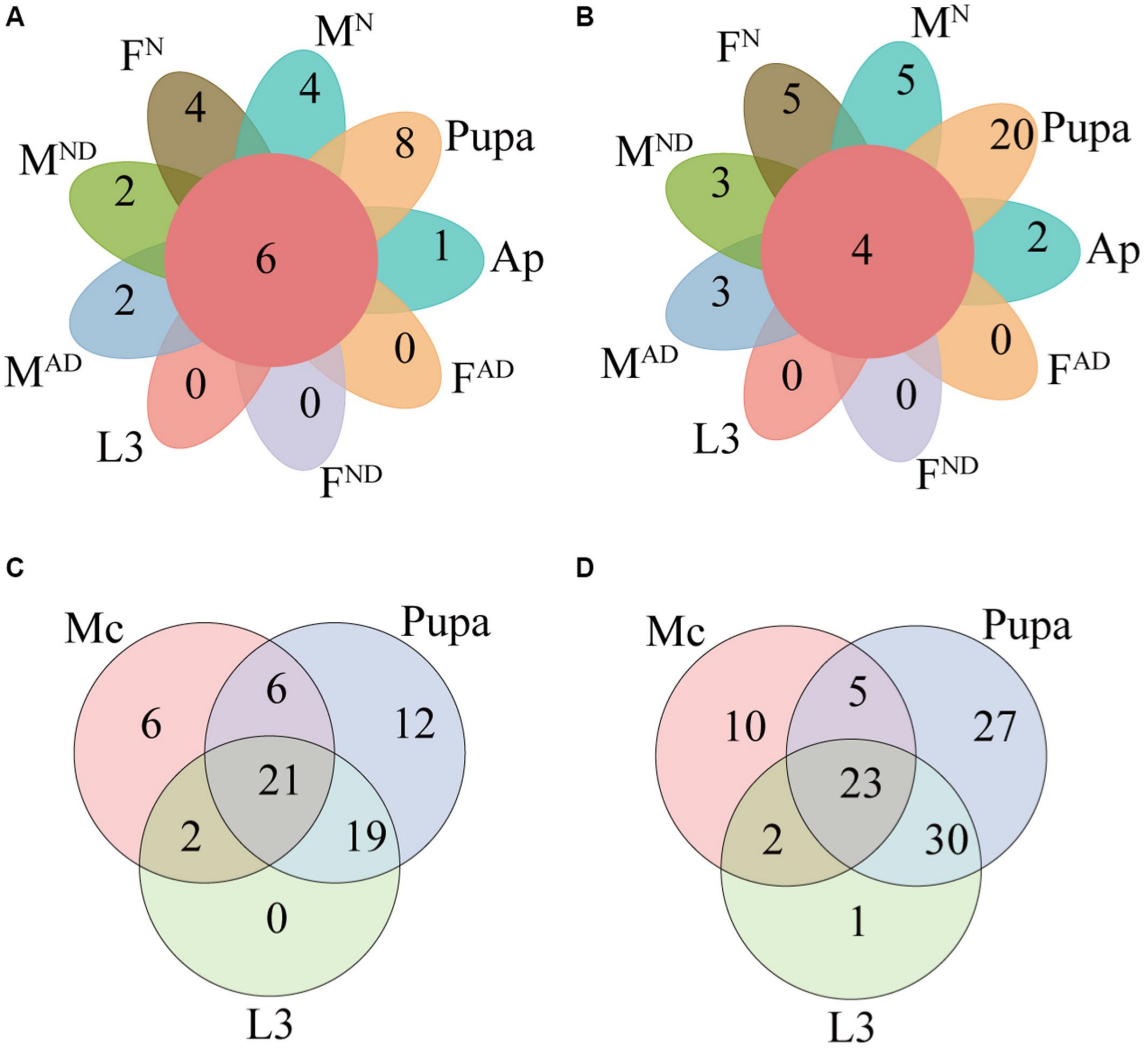
Petal diagram presenting the shared and unique bacteria in all groups at the genus **(A)** and species levels **(B)**. Venn diagram presenting the unique and shared symbionts between *Megoura crassicauda*, the larvae, and the pupae of *Episyrphus balteatus* at the genus **(C)** and species levels **(D)**. The definition of Mc, L3, F^N^ and M^N^, F^AD^ and M^AD^, F^ND^, and M^ND^ was the same as above in Figure 1.

Furthermore, we identified the shared and exclusive bacteria in all aphids and hoverfly samples at different taxonomic levels including four shared bacterial phyla (Actinobacteriota, Bacteroidota, Firmicutes, and Proteobacteria), five shared bacterial classes (Actinobacteria, Bacteroidia, Bacilli, Alphaproteobacteria, and Gammaproteobacteria), five shared bacterial orders (Micrococcales, Lactobacillales, Staphylococcales, Burkholderiales, and Enterobacterales), seven shared bacterial families (Microbacteriaceae, Enterococcaceae, Staphylococcaceae, Comamonadaceae, Erwiniaceae, Morganellaceae, and Yersiniaceae), six shared bacterial genera (*Enterococcus*, *Staphylococcus*, *Cedecea*, *Pantoea*, *Rosenbergiella*, and *Serratia*), and four shared bacterial species (*Staphylococcus xylosus*, *Cedecea neteri*, *Rosenbergiella nectarea*, and *Serratia symbiotica*; Supplementary Figure S2; Figures 4A,B; Supplementary Table S5).

To explore the potential horizontal transmission of symbionts across aphids and hoverflies resulting from the food chain, the microbiome from *M. crassicauda*, larvae, and pupae of *E. balteatus* were further analyzed in-depth at different taxonomic categories (Figures 4C,D; Supplementary Figure S3; Supplementary Table S5). A total of 23 bacteria species separately belonging to 21 genera, 18 families, 10 orders, 5 classes, and 5 phyla were shared by all these three group samples above. Those shared symbionts are probably horizontally transferred to hoverflies from aphids. A higher relative abundance of the aphid endosymbiotic bacterium *Serratia symbiotica* (30-fold) in hoverfly larvae compared to hoverfly pupae also demonstrates that horizontal transmission of symbionts likely occurs between hoverfly and prey aphids.

Compared to hoverfly larvae, 19 out of 23 bacteria species had a higher abundance of hoverfly pupae, especially in *Brevibacterium linens* (77-fold), *Brucella pseudogrignonensis* (55-fold), *Sphingobacterium multivorum* (39-fold), and *Staphylococcus xylosus* (32-fold). Interestingly, two bacteria species (*Hafnia alvei* and *Akkermansia muciniphila*) were shared only by *M. crassicauda* aphids and *E. balteatus* larvae but did not exist in hoverfly pupae (Figure 4D; Table S5), which hints their failure in the transmission through aphids to pupae. Although *Hafnia alvei* and *Akkermansia muciniphila* failed to colonize in the pupal stages of hoverflies, both can be detected in hoverfly adults (Figure 4; Supplementary Tables S4, S5). Hence, the transmission of bacteria across aphids and hoverflies is complicated.

In addition, compared to aphids and hoverfly larvae, 27 bacteria species were uniquely identified in hoverfly pupae, hinting at their colonization in pupae through the environment directly. Furthermore, 20 of these 27 bacteria species can also not be detected in any hoverfly adults, suggesting their important roles for pupae of hoverflies other than adults. The remaining 7 out of 27 bacteria species can be identified in some hoverfly adults, albeit with low abundance (Figure 4D; Supplementary Table S5), hinting at their low transmission efficiency through pupae to adults. Overall, further clarification is needed regarding how these 27 microbes colonize and work in the pupae of hoverflies in future studies.

### 3.4 Sexually biased symbionts in *Episyrphus balteatus*

**Figure 5:**
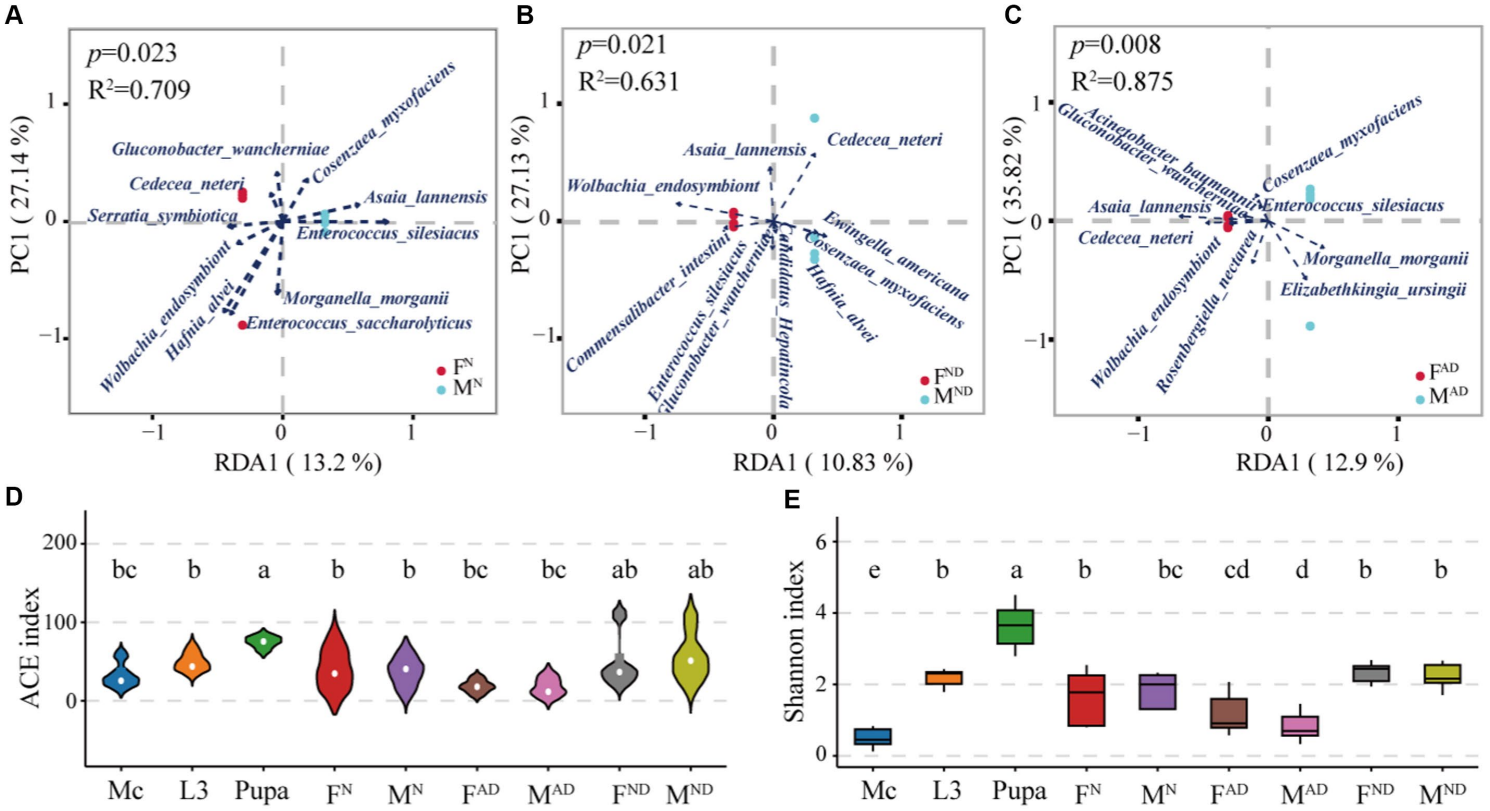
Identification of sexually biased symbionts from hoverflies and microbiota community diversity distribution of all groups. The interactions between gender and bacteria species in the pairwise comparison of F^N^ vs. M^N^ **(A)**, F^ND^ vs. M^ND^ **(B)**, and F^AD^ vs. M^AD^ **(C)**. The interactions of bacteria species in response to gender were illustrated via R package vegan (v2.3-0) with a cutoff of R^2^ > 0.6 and *p* < 0.05. R-square was used to evaluate the fitting degree of the model, and the *p*-value was used to evaluate whether gender (male or male) significantly influences the structure of biota. The larger the R-square, the better the goodness of degree fitting, and the smaller the *p*-value, the more significant the influence of gender on biota. Furthermore, the arrow length represents the correlation degree, and the intersection acute or obtuse angle between samples and the arrow starting point represents positive or negative correlations, respectively. A violin plot was used to show the symbiont community richness through the ACE index **(D)**, and the community diversity was exhibited with a boxplot measured by the Shannon index **(E)**. Different lowercase labels above each group indicate significant differences (Wilcoxon test, *p* < 0.05) in group mean value.

In line with β-diversity, redundancy analysis (RDA) was used to evaluate the correlation between microbiota composition and hoverfly gender at the species level (Figure 4). The results indicated that most normal female hoverflies had positive correlations with four bacteria species (*Wolbachia_endosymbiont_of_Bryobia_praetiosa*, *Serratia symbiotica*, *Cedecea neteri*, and *Gluconobacter wancherniae*), while most normal male hoverflies had positive correlations with three bacterial genera (*Enterococcus silesiacus*, *Asaia lannensis*, and *Cosenzaea_myxofaciens_ATCC_19692*; Figure 5A). For normally dying female hoverflies, their positive related bacteria species were *Wolbachia_endosymbiont_of_Bryobia_praetiosa*, *Asaia lannensis*, *Commensalibacter intestini*, *Enterococcus silesiacus*, and *Gluconobacter wancherniae*. For normally dying male hoverflies, *Hafnia alvei*, *unclassified_Candidatus_Hepatincola*, *Cosenzaea_myxofaciens_ATCC_19692*, and *Ewingella americana* were the positive related bacteria species (Figure 5B). Similar results were also observed between abnormally dying newly emerged female and male hoverflies. The former had highly positive correlations with three bacteria species *Asaia lannensis*, *Cedecea neteri*, and *Gluconobacter wancherniae*, followed by *Wolbachia_endosymbiont_of_Bryobia_praetiosa*, *Acinetobacter guillouiae*, and *Rosenbergiella nectarea*. On the contrary, the latter had highly positive correlations with the bacteria species *Enterococcus silesiacus, Morganella morganii, and Elizabethkingia ursingii* (Figure 5C).

Although the relative abundances of those bacteria species above were sexually biased with hoverfly gender, there were no significant differences in microbiota community richness and diversity across those pairwise comparisons of F^N^ vs. M^N^, F^AD^ vs. M^AD^, and F^ND^ vs. M^ND^ (Figures 5D,E). Interestingly, the immobile and non-feeding hoverfly pupae had the highest bacterial community richness and diversity, followed by hoverfly larvae, and *M. crassicauda* had the lowest.

### 3.5 Co-occurrence of microbial community and biomarker identification

**Figure 6:**
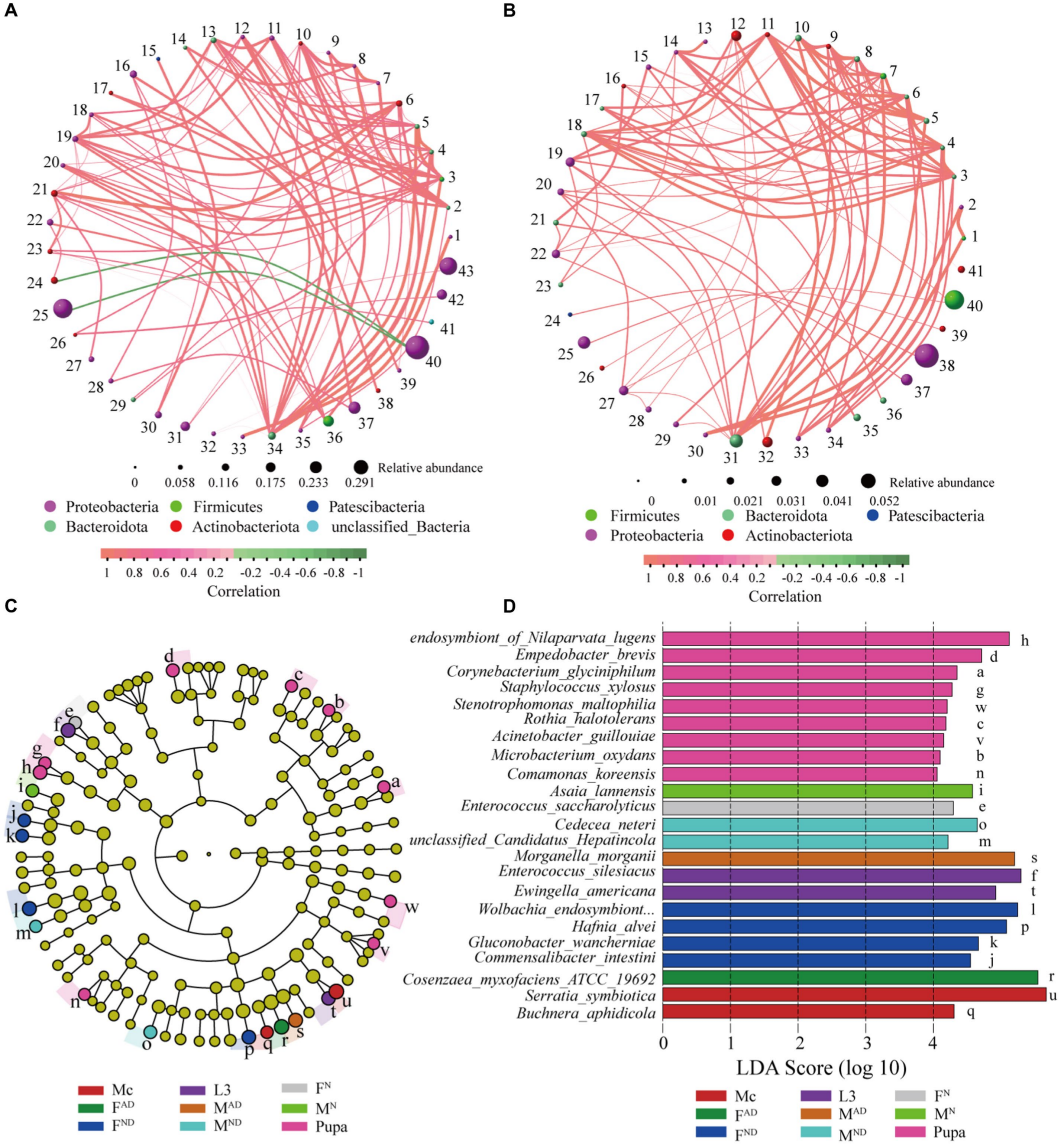
Co-occurrence network diagram of pairwise symbionts and biomarkers identified through LEfSe analysis. Co-occurrence patterns of pairwise symbionts at the genus **(A)** and species **(B)** levels in the hoverfly. The one-to-one correspondence of sequence numbers (1 to 43/40 in Figures 5A,B) and bacterial genus or species are shown in Supplementary Table S7. The sphere nodes represent genus **(A)** or species **(B)**, and the sphere size represents the relative abundance. The sphere color represents different phylum levels to which genus **(A)** or species **(B)** belonging to. The line represents the correlation between the two genera **(A)** or species **(B)**, in which the thickness of the line represents the strength of the correlation and the color of the line: red represents the positive correlation, and green represents the negative correlation. Cladogram of bacterial taxa among samples by LDA effect size analysis **(C)**. In the cladogram, the circles from the inside to the outside denote the taxonomic level from phylum to species level, and the diameter of the small circle is proportional to the relative abundance. Different species are colored according to the highest abundance group with lowercase letters which was explained in **(D)**. The eligible biomarkers were identified based on the criteria of LDA score > 4 **(D)**. Different colors represent the biomarkers from different groups.

A network correlation analysis was conducted to explore the co-occurrence pattern of symbionts from aphids and hoverfly samples at the genus and species level, respectively. As shown in Figure 6A, 43 nodes (bacteria genera) and 100 edges are observed in the network analysis (R > 0.7, *p* < 0.01) at the genus level, while 41 nodes (bacteria species) and 100 edges are observed in the network analysis at the species level (R > 0.7, *p* < 0.01; Figure 6B). LEfSe analysis was used to identify the most differentially abundant bacteria (biomarkers) in each group from aphids and hoverflies at the species level, and a total of 23 biomarkers (LDA score > 4) were obtained (Figures 6C,D). For *M. crassicauda* aphids, *Serratia symbiotica* and *Buchnera aphidicola* were the most abundant bacteria species, which can be used as distinguishing biomarkers. For larvae of hoverflies, the predominant bacteria *Enterococcus silesiacus* and *Ewingella americana* are the biomarkers. In addition, pupae of the hoverfly had the largest quantity (9) of bacteria species as biomarkers including *endosymbiont_of_Nilaparvata_lugens*, *Empedobacter brevis*, *Corynebacterium glyciniphilum*, *Staphylococcus xylosus*, *Stenotrophomonas maltophilia*, *Rothia halotolerans*, *Acinetobacter guillouiae*, *Microbacterium oxydans*, and *Comamonas koreensis*. For normal female and male hoverfly adults, *Enterococcus saccharolyticus* and *Asaia lannensis* were the biomarkers, respectively. Furthermore, four bacteria species *Wolbachia_endosymbiont_of_Bryobia_praetiosa*, *Hafnia alvei*, *Gluconobacter wancherniae*, and *Commensalibacter intestine* could be used as biomarkers for FD^N^ hoverfly adults, while *Cedecea neteri* and *unclassified_Candidatus_Hepatincola* are biomarkers for MD^N^ hoverfly adults. Finally, for F^AD^ and M^AD^ hoverfly adults, *Cosenzaea_myxofaciens_ATCC_19692* and *Morganella morganii* were the biomarkers, respectively.

### 3.6 Microbiota function prediction and comparison

**Figure 7:**
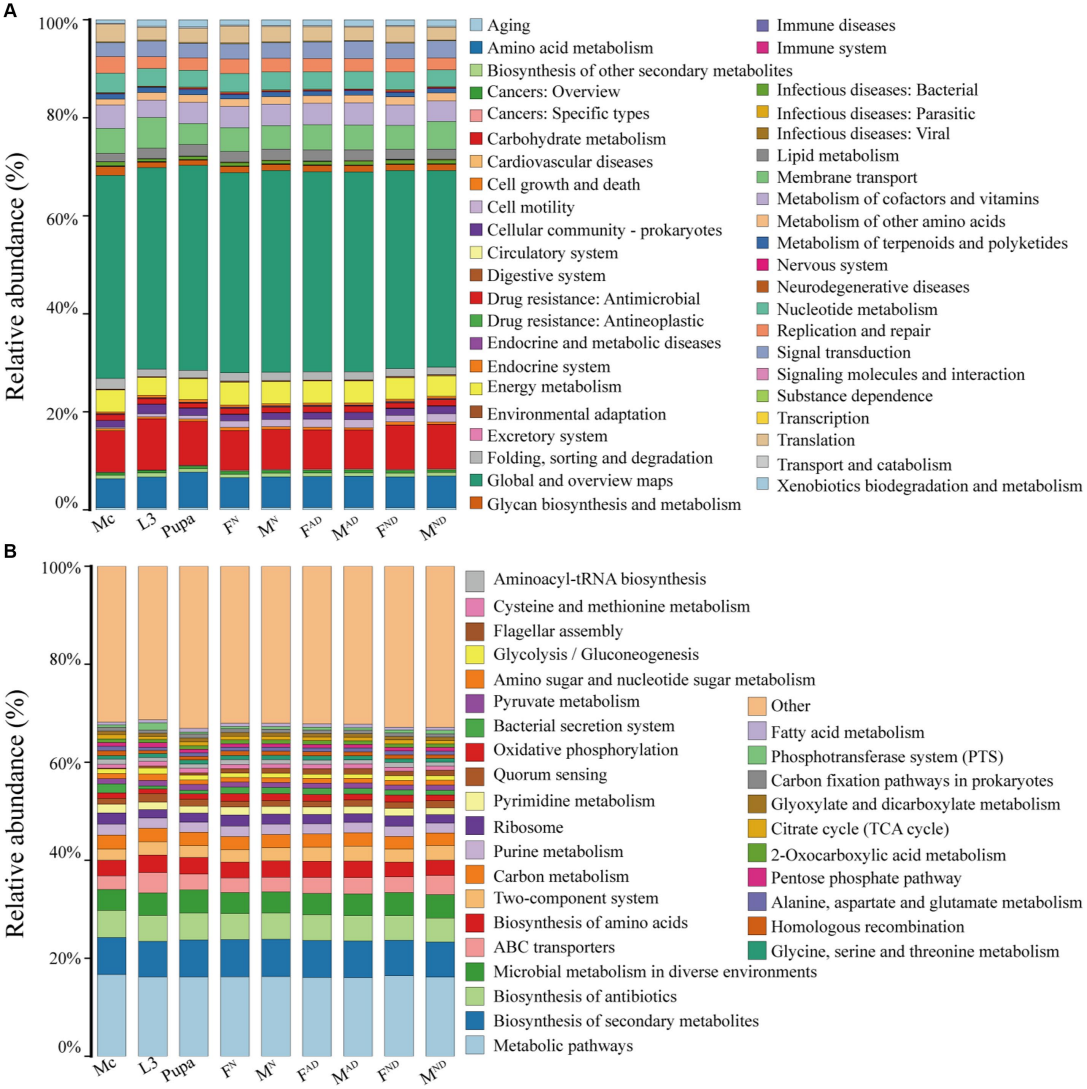
Functional pathway abundance prediction of bacteria in all hoverflies and aphids against KEGG categories at level 2 **(A)** and level 3 **(B)** by PICRUSt2. At level 2, pathways ranked below 30 in total relative abundance were classified as “Others”.

The biological functions of the microbiota of aphids and hoverflies were predicted using PICRUSt2 software based on the full-length sequences of 16S rRNA by comparing against the KEGG database. The results were demonstrated at two levels (Figure 7). In level 2, 43 categories of biological functions were obtained, in which the relative abundance of global and overview maps was the highest (40.1%–41.8%) in all samples, followed by carbohydrate metabolism (7.9%–10.4%), amino acid metabolism (5.9%–7.2%), membrane transport (4.3%–6.3%), energy metabolism (3.6%–4.6%), and nucleotide metabolism (3.4%–4.0%; Figure 7A). In level 3, the richness of the top 30 pathways was shown, and the other pathways are classified as Others (Figure 7B). The relative abundance of metabolic pathways (16.1%–16.7%) was the highest in all aphids and hoverfly samples. In addition, pathways related to the biosynthesis of secondary metabolites (7.1%–7.6%) and antibiotics (4.9%–5.5%) account for the vast majority, and pathways involved in microbial metabolism in diverse environments, ABC transporters, biosynthesis of amino acids, and carbon metabolism were abundantly enriched as well. Interestingly, although the microbiota structure and diversity were distinct among samples from aphids and hoverflies (Figure 3; Supplementary Figure S1), the enriched biological function categories of microbiota from aphids and hoverflies were both similar at level 2 and level 3 (Figure 7).

**Figure 8:**
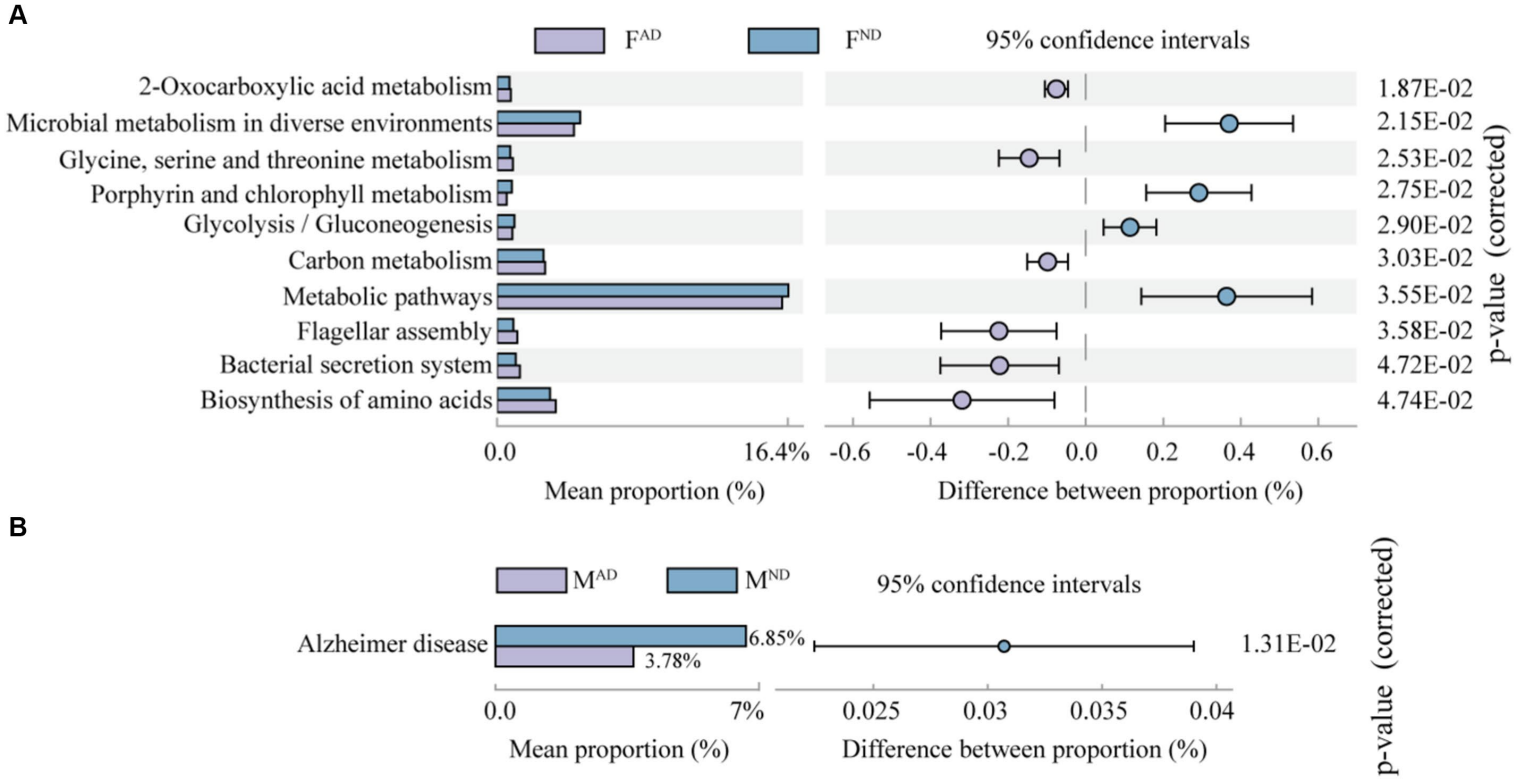
Inferred function comparison of symbiont communities between abnormally dying newly emerged hoverflies and normally dying hoverflies [F^AD^ vs. F^N^ **(A)**; M^AD^ vs. M^ND^ **(B)**] at level 3.

In addition, the abundance differences of the KEGG pathway were identified in the pairwise comparison of abnormally dying newly emerged adults vs. normally dying adults, normally dying adults vs. normal living adults, and abnormally dying newly emerged adults vs. normally living adults. Interestingly, no significant difference in the KEGG pathway existed in the latter two pairwise comparisons. In comparison to F^ND^ hoverflies, F^AD^ had a higher abundance of pathways related to 2-oxocarboxylic acid metabolism, “glycine, serine, and threonine metabolism,” carbon metabolism, flagellar assembly, bacterial secretion system, and biosynthesis of amino acids. However, it exhibited a lower abundance of pathways relative to microbial metabolism in diverse environments, porphyrin and chlorophyll metabolism, glycolysis/gluconeogenesis, and metabolic pathways (Figure 8A). In addition, the abundance of pathway Alzheimer’s disease was significantly higher in M^ND^ than in M^AD^ (Figure 8B). Alzheimer’s disease is one of the common neurodegenerative diseases in many animals other than just humans, although it mostly affects the elderly population of people (Tsuda and Lim, 2018; Ulian-Benitez et al., 2022). Although a causal link between Alzheimer’s disease pathway and hoverflies remains elusive and does not seem relevant, the well-known model “microbiota–gut–brain axis” may help understand their relationship. Emerging evidence indicates that gut dysbiosis may promote amyloid-beta aggregation, neuroinflammation, oxidative stress, and insulin resistance in the pathogenesis of Alzheimer’s disease (AD; Liu et al., 2020). Compared to M^ND^, a higher abundance of pathway Alzheimer’s disease enriched from the symbiont in M^AD^ hoverfly probably contributes to the relatively high mortality of newly emerged adults (Figure 1A) caused by disordered functional microorganisms.

## 4 Discussion

In this study, we systematically investigated the microbiome structure of one Syrphidae hoverfly shaped by developmental stages, living states, and two sexes. Proteobacteria and Firmicutes, two predominating bacteria phyla existing in most insect taxa and metabolizing phytochemicals for hosts (Xue et al., 2021), were also dominant in all *E. balteatus* samples. Gammaproteobacteria was the primary bacteria class in all hoverfly samples, which is consistent with reports in the striped shield bug *Graphosoma Lineatum* (Karamipour et al., 2016). In order level, except for pupae and F^ND^ adults, the predominant microbe is Enterobacterales, which was often reported as the most abundant bacterial community in many kinds of insects (Zhao et al., 2019; Xue et al., 2021; Xu et al., 2023). Overall, dominant symbiont categories in *E. balteatus* are similar to those in other insect taxa.

Interestingly, at the family level, Morganellaceae is the most dominant bacteria in hoverfly adults with a relative abundance of 53.7%–91.65%. However, the relative abundance of Morganellaceae in hoverfly larvae was only 7.1%, and Yersiniaceae is the predominant bacteria in the larvae of the marmalade hoverfly. We speculated that this difference is due to their distant feeding habits because *E. balteatus* larvae and adults were fed on *M. crassicauda* aphids and 2% sucrose solution with pollen, respectively. The predominant bacteria Yersiniaceae in aphids probably horizontally transmit from aphids into larvae of hoverflies and successfully turn into the dominant bacteria as well. Interestingly, Morganellaceae has been reported to occur exclusively as symbionts of Rhabditida nematodes, for which they can kill insects and provide nutrition and defense within the cadavers (Maher et al., 2021; Ulug, 2023). Hence, we speculated that *E. balteatus* can be infected by some nematodes as other Syrphidae (Diptera) insects. For example, two *Skarbilovinema entomoparasitic* nematodes can parasite hoverflies from the genus *Helophilus* and *Eristalis* of the family (Chizhov et al., 2012). In another Diptera insect, human-biting black fly *Simulium nigrogilvum* was commonly parasitized by diverse nematodes (Huang et al., 2021).

In genus and species levels, *Cosenzaea* (*C. myxofaciens ATCC_19692*) was the most dominant bacteria in most hoverfly samples with a relative abundance of 51.2%–68.1%. The genus *Cosenzaea* belongs to the family of Enterobacteriaceae which comprises only one species *C. myxofaciens* that was originally isolated from the larva of a gypsy moth (Ventorino et al., 2016). However, regrettably, knowledge of the roles of *C. myxofaciens* in insects remains little. *C. myxofaciens* was named *Proteus myxofaciens* previously and was transferred into a novel genus *Cosenzaea* in 2011 (Giammanco et al., 2011) which still consists of this single species (Behrendt et al., 2019). Genome-based phylogeny of the 13 species of *Proteus* and *Cosenzaea* suggests that the only species (*C. myxofaciens*) of the genus *Cosenzaea* belongs to the genus *Proteus* actually (Behrendt et al., 2019). *Proteus* genus bacteria are known to be human opportunistic pathogens, and many wild and domestic animals are the hosts of *Proteus* spp. bacteria (Drzewiecka, 2016). *Proteus* spp. were proved to be the most common bacteria among gram-negative and lactose-negative ones isolated from flies [i.e., hematophagous sand fly *Phlebotomus papatasi* (Maleki-Ravasan et al., 2014), *Lucilia sericata* (Ma et al., 2012), the green bottle fly *Lucilia cuprina* (Singh et al., 2015), and the housefly *Musca domestica* (Wei et al., 2014)], of which several spp. Were usually the dominating genus in some flies (Drzewiecka, 2016). The *Proteus* spp. Abilities to metabolize various toxic compounds and unusual physiological features (i.e., cellulose digestion, hydrocarbons utilization, and lipase production) contribute to the symbiosis between flies and *Proteus* bacteria (Drzewiecka, 2016). In this study, whether dominant symbionts *Cosenzaea* identified in most marmalade hoverfly adults are human opportunistic pathogens deserves careful study in the future.

*Wolbachia* is the widespread bacterial endosymbiont among arthropod species with a 19 to 76% infection rate and with the broadest range of host reproductive phenotypes, including induction of cytoplasmic incompatibility, feminization of genetic males, parthenogenesis, and male-killing (Sasic Zoric et al., 2019). Several attempts were made to test for the presence of *Wolbachia* in hoverfly species (Syrphidae). *Graptomyza brevirostris* (Eristalinae: Volucellini) tested positive for both *ftsZ* and *Wolbachia* surface protein (*wsp*) genes (Sintupachee et al., 2006), and three Eristalinae hoverfly species (*Rhingia campestris*, *Eristalis arbustorum,* and *E. tenax*) were all positive for the tested *Wolbachia CoxA* gene primers (Evison et al., 2012). ZORIC first reported that bacterial endosymbiont *Wolbachia* existed in 50 out of 54 (96% infection rate) phytophagous hoverfly species from the genus *Merodon* (Sasic Zoric et al., 2019). In addition, *Wolbachia* was also detected in other pollinators such as honey bees, bumblebees, and wasps (Evison et al., 2012). Studies from Sintupachee (Sintupachee et al., 2006) and ZORIC (Sasic Zoric et al., 2019) both suggest that plant-mediated horizontal transmission of *Wolbachia* among hoverfly species existed. Our study supported their opinions: *Wolbachia endosymbiont* was not tested in the prey *M. crassicauda* aphids but only in *E. balteatus*. Although its relative abundance is low in marmalade hoverfly larvae (0.3%), it then increases to 4% in pupae and peaks at 19.6–28.6% in normal adults as the second dominant symbiont species.

In attempt of exploring potential sources of symbiotic bacteria, it was found that *Serratia symbiotica*, a facultative endosymbiont commonly harbored in most aphid species, dominating in *M. crassicauda*, was also detected in all *E. balteatus* samples, which is the second dominant in larvae of marmalade hoverfly. This result suggests that the aphid symbiont *S. symbiotica* probably potentially horizontally transmitted to *E. balteatus* from *M. crassicauda*, which is in agreement with reports from Du in aphidophagous ladybirds which obtain *S. symbiotica* through prey aphids (Du et al., 2022). Compared to non-aphidophagous ladybirds (*Henosepilachna vigintioctopunctata* and *Cryptolaemus montrouzieri*), aphidophagous (aphid-feeding) ladybirds (*Propylea japonica*, *Coccinella septempunctata*, *Micraspis discolor*, *Cheilomenes sexmaculata*) have evolved to establish a nearly neutral relationship with *S. symbiotica* horizontally transmitted from prey aphids, suggesting adaptation to a prey symbiont (Du et al., 2022). In addition, *Serratia* was also enriched in larvae and pupae of aphid-feeding *Chrysoperla sinica* (Zhao et al., 2019). Several studies are accumulating evidence that endosymbionts of phytophagous insects transmit horizontally via plants, subsequently transferring them to their progeny (Chrostek et al., 2017). Although our study provides insight into the possibility of the transmission of symbiosis *Serratia* between prey aphids and predator hoverflies, more experimental evidence should be provided to validate this speculation in the future.

In addition, in our study, the dominant symbiont *M. crassicauda* was unexpectedly *S. symbiotica* (a facultative endosymbiont commonly harbored in most aphid species) with a relative abundance of 92.1%, other than *Buchnera aphidicola* (the familiar primary symbiotic bacterium inhabited in most aphid species), just accounting for 4.7% of relative abundance. The aphid microbiota can be shaped by aphid species, geography, and host plants. Qin et al. characterized the microbial compositions of 215 aphid colonies representing 53 species of the aphid subfamily Greenideinae of Aphididae from different regions and plants in China, Nepal, and Vietnam. They also found that primary endosymbiont *Buchnera* was present in all the samples and predominated in most species. Furthermore, *S. symbiotica* was the most abundant secondary symbiont in Greenideinae; however, its relative abundance was even higher than that of *Buchnera* in the aphid genus *Schoutedenia* (Qin et al., 2022). Similarly, whether *S. symbiotica* was the most dominant bacterial species in wild *M. crassicauda* population needs to be validated in the nest study. In addition, the microbiota structure of insects is known to be influenced by laboratory-rearing conditions or seasons; although some genera are stably maintained and can be consistently detected irrespective of those conditions, the majority of microbiota remain varied (Suenami et al., 2023). Hence, the unexpected relative abundance of *Serratia* and *Buchnera* is likely due to rearing the hoverflies in the laboratory for many generations. Similarly, Cambon et al. found that changes in rearing conditions can rapidly modify the microbiota structure in *Tenebrio molitor* larvae (Cambon et al., 2018). Waltmann et al. found that, although the microbiota of laboratory-reared *Triatoma infestans* comprised a subset of those identified in their wild counterparts, the bacterial diversity in wild *T. infestans* was greater than in laboratory-reared bugs (Waltmann et al., 2019). In addition, we cannot rule out the influence of host plant species on microbiota structure of insects. For example, the microbiome structure of the aphid *Myzus persicae* can be shaped by different plant diets (He et al., 2021). Compared to aphids feeding on cabbage, a substantial increase in the abundance of *Pseudomonas* was observed in individuals that fed on eggplant and tobacco, accounting for up to 69.4% of the bacterial community in *M. persicae*, with a substantial decrease in the abundance of the primary symbiont *Buchnera* (He et al., 2021).

## 5 Conclusion

The present study conducted a detailed investigation of *Episyrphus balteatus* microbiota dynamics across developmental stages (larvae, pupae, adults), different living states (adults of health, normally dying, newly emerged dying), and two sexes for the first time. Additionally, it explored the potentially horizontal transmission of symbionts from prey *Megoura crassicauda* (i.e., *Serratia symbiotica*) or the environment (i.e., *Wolbachia endosymbiont*) to *E. balteatus*.

The dominant symbiont phyla of *E. balteatus* were Proteobacteria and Firmicutes, but their relative abundance fluctuated by the host developmental stage. A potentially human opportunistic pathogen *Cosenzaea myxofaciens* seen in other insects is unexpectedly dominant in marmalade hoverfly adults and is noteworthy to pay attention to it in future. Furthermore, numerous symbiont species were sexually biased between two sexes of *E. balteatus*, and 41 microbial species pairwise have co-occurrence relationships. Functional prediction showed symbiotic bacteria of hoverflies and aphids, both mainly focusing on metabolic pathways. Overall, using *E. balteatus* as a model, we first explored hoverfly symbionts’ variation across developmental stages, living states, two sexes, and potential horizontal transmission from aphids. The results provide basis for future studies elucidating symbionts’ roles in hoverflies.

## Data availability statement

The datasets presented in this study can be found in online repositories. The names of the repository/repositories and accession number(s) can be found at: https://www.ncbi.nlm.nih.gov/, PRJNA1020272.

## Ethics statement

The manuscript presents research on animals that do not require ethical approval for their study.

## Author contributions

XC: Methodology, Software, Validation, Writing – original draft, Writing – review & editing, Data curation, Formal Analysis, Investigation, Resources, Visualization. SX: Conceptualization, Resources, Validation, Writing – review & editing. RL: Formal Analysis, Methodology, Writing – review & editing. YZ: Conceptualization, Funding acquisition, Project administration, Supervision, Validation, Writing – review & editing.

## Footnotes

^1^http://www.biocloud.net/
