# Supplementary figures and images for: Episyrphus balteatus symbiont variation across developmental stages, living states, two sexes, and potential horizontal transmission from prey or environment
Source: Front Microbiol. 2024 Jan 5;14:1308393. doi: 10.3389/fmicb.2023.1308393 (PMC10797133; doi:10.3389/fmicb.2023.1308393)

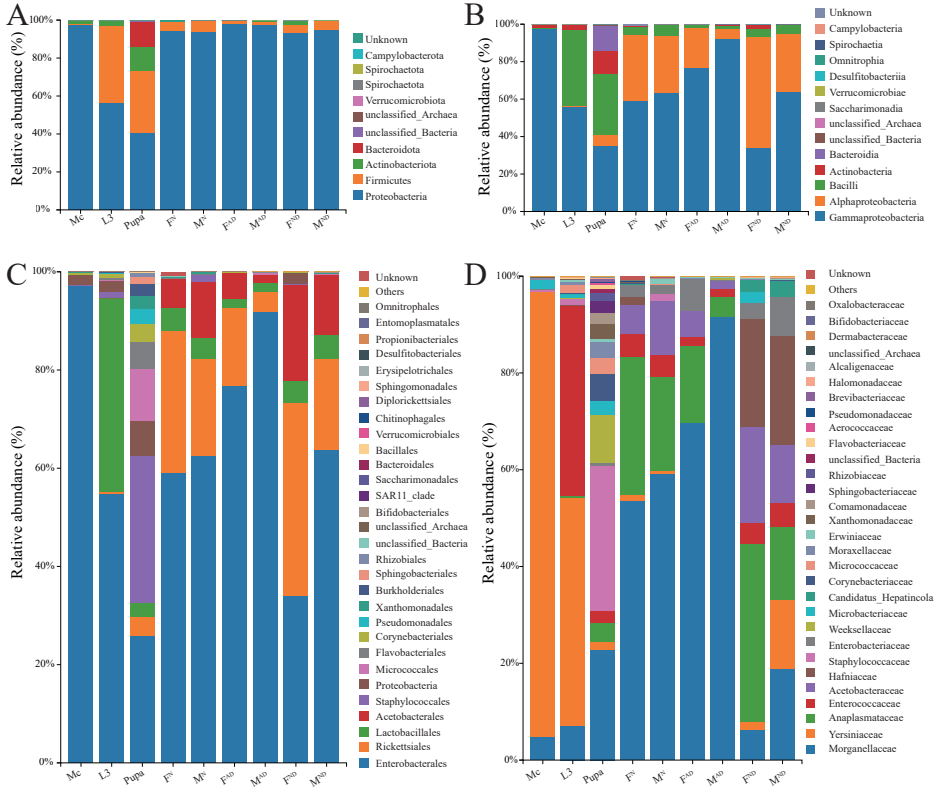

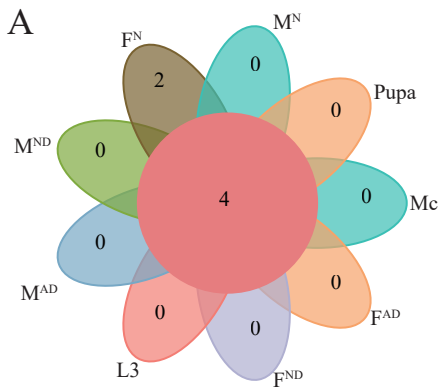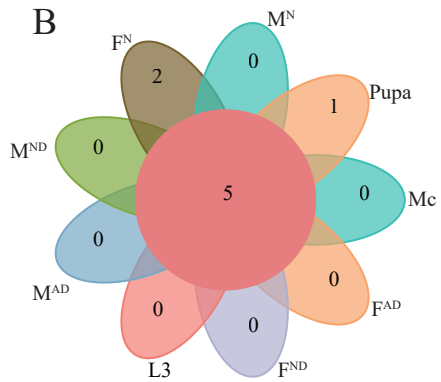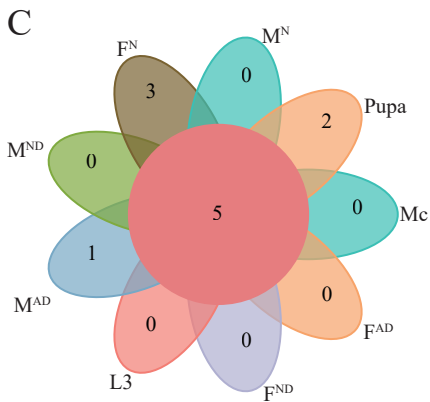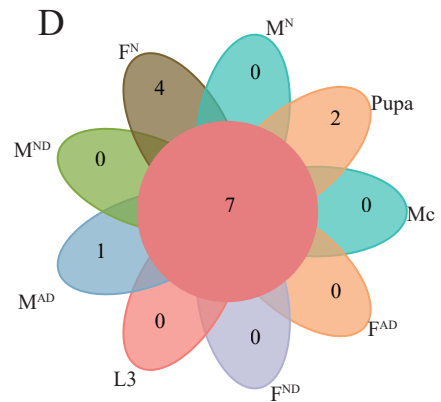

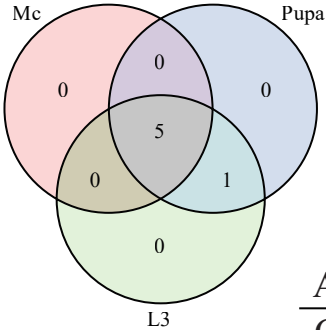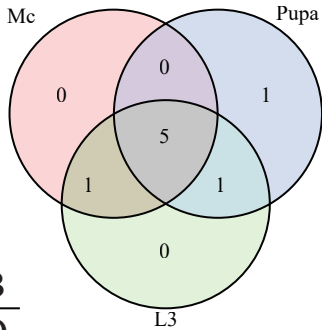

$$\frac{A|B}{C|D}$$

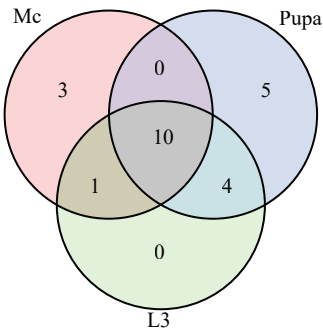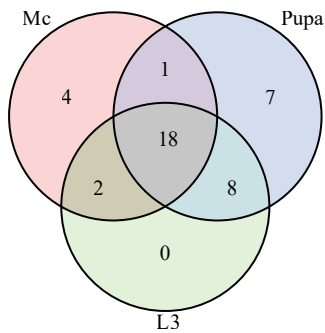

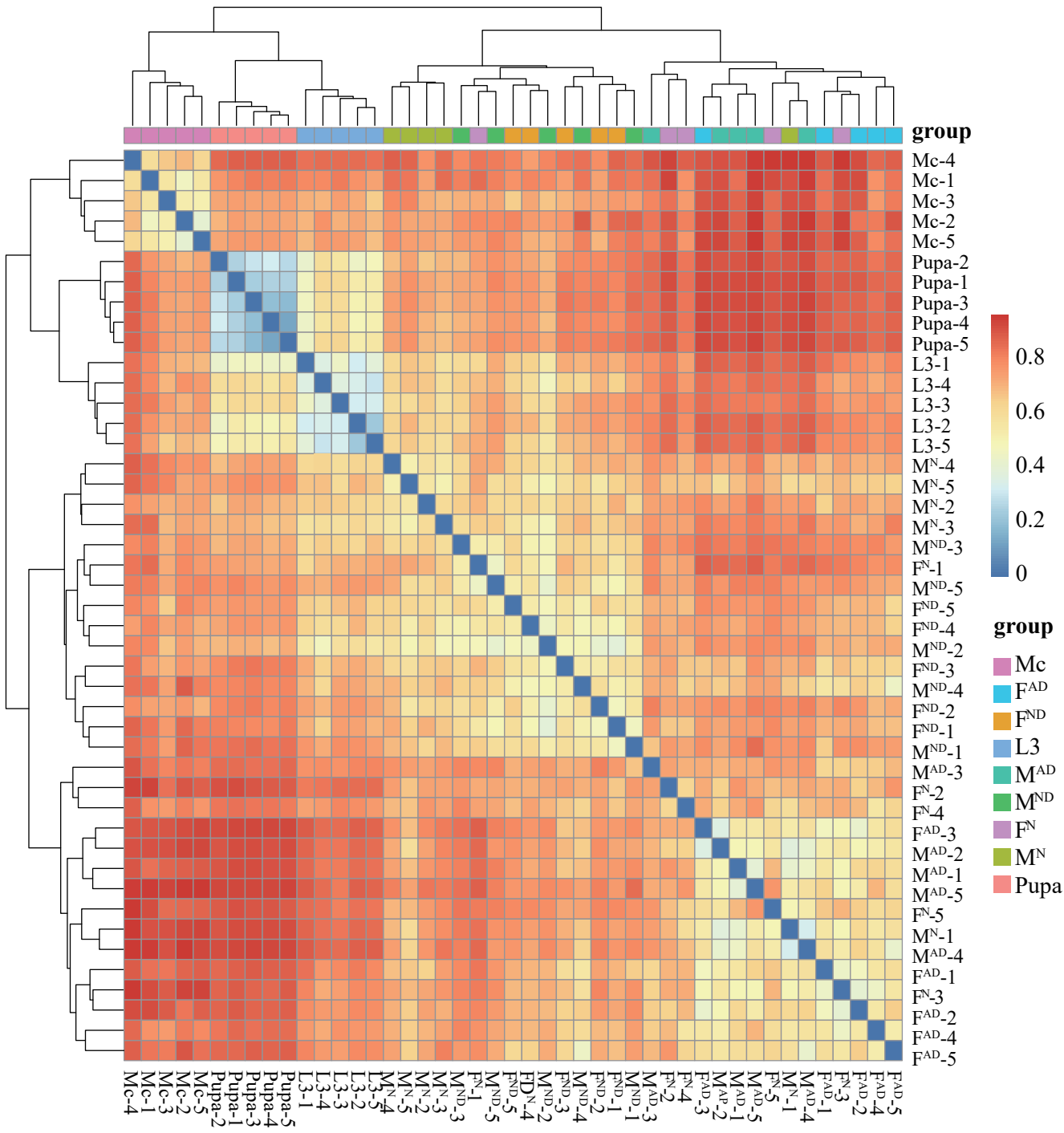

Supplement: Supplementary file 9 [file Data_Sheet_1.pdf]
